# Production of β‐carotene with *Dunaliella salina* CCAP19/18 at physically simulated outdoor conditions

**DOI:** 10.1002/elsc.202000044

**Published:** 2020-09-13

**Authors:** Lara Wolf, Thomas Cummings, Katharina Müller, Manfred Reppke, Marianne Volkmar, Dirk Weuster‐Botz

**Affiliations:** ^1^ Institute of Biochemical Engineering Technical University of Munich Garching Germany

**Keywords:** *Dunaliella salina*, increasing salinity, outdoor conditions, photobioreactor, ß‐carotene

## Abstract

Batch growth and β‐carotene production of *Dunaliella salina* CCAP19/18 was investigated in flat‐plate gas‐lift photobioreactors with a light path of 2 cm, operated in physically simulated outdoor conditions. *Dunaliella salina* CCAP19/18 showed robust growth with respect to pH 8.0‐9.0 and 15–35°C at increasing salinity, simulating the evaporation of open photobioreactors. The highest β‐carotene concentration of 25 mg L^‐1^ (3 mg g_CDW_^−1^) was observed in batch processes at pH 8.5, 15–30°C and increasing salinity up to 110 g L^‐1^, simulating a typical Mediterranean summer climate. Intracellular β‐carotene accumulation of *D. salina* CCAP19/18 was shown to be independent of light availability, although nutrient limitation (K_2_HPO_4_, MgSO_4_, and/or ammonium ferric citrate) seems to enable stable β‐carotene content in the algal cells despite increasing cell densities in the photobioreactor. Fully controlled, lab‐scale photobioreactors simulating typical climate conditions of any region of interest are valuable tools for enabling a realistic characterization of microalgae on a laboratory scale, for production processes projected in open photobioreactor systems (e.g. thin‐layer cascade photobioreactors).

## 1 INTRODUCTION

CO_2_‐neutral sources of chemicals are becoming increasingly popular due to rising CO_2_ emissions and the resulting global warming [1]. Microalgae are one promising alternative to fossil resources, as they use CO_2_ and light for growth [2, 3, 4]. Microalgae enable higher solar energy yields compared to plants, and can be produced in suitable climate zones throughout the whole year [5, 6, 7, 8] making use of seawater or wastewater [9, 10]. If non‐arable land is used for the production of microalgae biomass, there will be no competition with agriculture [11].

β‐carotene is a tetraterpene with eleven conjugated double bonds. Thus, it is able to absorb visible light and appears red‐orange [12]. In photosynthetic active cells, it serves as an accessory pigment, and offers protection from oxidation reactions [13]. β‐carotene can be used as a colorant, food and feed additive or as precursor for the production of aromatic antioxidants for aviation fuels [14, 15, 16]. Even though the all‐*trans* form of β‐carotene can be synthesized chemically [17], there are advantages of the naturally produced β‐carotene composition, such as its higher antioxidative capacities [15]. The global market value of β‐carotene in 2015 was USD 432.2 million, whereby USD 164.2 million worth of this β‐carotene was extracted from algae [18].

*Dunaliella salina*, a photoautotrophic and halotolerant microalga, is known for the ability to accumulate β‐carotene. As *D. salina* tolerates salinities up to saturation concentrations > 300 g L^−1^ NaCl [19], it is particularly suitable for outdoor cultivations with seawater, resulting in increasing salinity due to evaporation. The large‐scale production of *D. salina* is carried out either in shallow lagoons without mixing or in raceway ponds mixed with paddle wheels [20].

β‐carotene accumulation in *D. salina* can be induced by increasing salinity, increasing photon flux density or nutrient limitations [21, 22, 23]. Final carotenoid concentrations of 8.6 mg L^‐1^ and 21 mg L^‐1^ have been reported with *D. salina* CCAP19/18 cultivated in shaking flasks [24], and Roux bottles [25], respectively. This corresponds well to the carotenoid concentrations of 19–20 mg L^‐1^ measured in open raceway pond cultivations of *D. salina* at cell dry weight concentrations of 0.33–1.2 g_CDW_ L^‐1^ [26, 27]. Increasing the final biomass concentrations of *D. salina* in the photobioreactor would be beneficial in terms of reducing the costs for isolation of the intracellular β‐carotene [28]. As raceway ponds limit algal growth due to high culture depths and resulting light limitations, open systems with a much smaller culture depth can be an alternative. Open thin‐layer cascade photobioreactors enable cell dry weight concentrations of up to 50 g_CDW_ L^‐1^ with a culture depth of only 6 mm [29, 30]. In these studies the scale up of laboratory scale processes performed in flat‐plate photobioreactors to the pilot scale operated in thin‐layer cascade reactors could be shown with the scale‐up criterion ’mean integral photon flux density’ despite the differences in geometry and power input. A detailed description and table of the geometric differences can be found in [29].

A high β‐carotene content of 27 mg g_CDW_^−1^ was reported with *D. salina* applying a flat panel photobioreactor operated as turbidostat at constant incident photon flux densities of 200 μmol m^‐2^ s^‐1^ and nitrogen depletion [31]. So far, growth and carotenoid production with *D. salina* was studied on a laboratory scale keeping all state variables constant, e.g. light intensity, temperature and salinity [32, 33, 34, 35, 36]. However, outdoor conditions are subject to day and night cycles of incident light and temperature. If seawater is used for the cultivation of *D. salina*, an increase of salinity is inevitable due to the evaporation of water.

Consequently, day‐night cycles imitating Mediterranean climate conditions in terms of light and temperature will be applied in this study to investigate growth and β‐carotene production of the strain *D. salina* CCAP19/18 given increasing salinity and varying pH set‐points. Fully controlled, flat‐plate gas‐lift photobioreactors will be used in parallel on a lab‐scale because the possibility of scaling up microalgal cultivation processes from flat‐plate bioreactors to open thin‐layer cascade reactors has already been demonstrated [29]. Increasing salinity as a consequence of evaporation compensation with seawater in open thin‐layer cascade reactors will be simulated by NaCl additions, based on published evaporation rates as a way of applying a typical Mediterranean summer climate [30].

**PRACTICAL APPLICATION**

Outdoor cultivations of microalgae are subject to variations of temperature and incident photon flux densities due to day and night cycles. As evaporation occurs due to the usual unsaturated air humidity, an increase in salinity in the water phase of the photobioreactor is unavoidable during long‐term operation. Unfortunately, most studies on the process performances of microalgae keep these important state variables constant, for example with studies of the halotolerant microalga *Dunaliella salina*, known as a producer of ß‐carotene, which is used industrially as a colorant, food and feed additive. Consequently, day‐night cycles imitating typical Mediterranean climate conditions in terms of light and temperature were applied in this study to investigate growth and β‐carotene production of the strain *D. salina* CCAP19/18 in well‐defined, flat‐plate gas‐lift photobioreactors at increasing salinity, simulating the evaporation of open photobioreactors, and thus enabling a more realistic characterization of the process performance of this microalgal strain.

## 2 MATERIALS AND METHODS

### 2.1 Microalgal strain and seed culture preparation

The photoautotrophic strain *D. salina* CCAP19/18 was obtained from the Culture Collection of Algae and Protozoa (CCAP) in Scotland. Shaking flask cultivations were used for strain maintenance at continuous photosynthetic active irradiation of 83±17 μmol m^−2^ s^−1^ between 400 and 750 nm and a constant temperature of 25°C with BG11 medium [37]: 50.0 g L^‐1^ NaCl; 1.5 g L^‐1^ NaNO_3_, 0.04 g L^‐1^ K_2_HPO_4_, 0.075 g L^‐1^ MgSO_4_ · 7 H_2_O, 0.036 g L^‐1^ CaCl_2_ · 2 H_2_O, 0.006 g L^‐1^ citric acid, 0.001 g L^‐1^ Na‐EDTA, 0.006 g L^‐1^ ammonium ferric citrate, 0.02 g L^‐1^ Na_2_CO_3_ and 1.0 mL L^−1^ trace solution A‐5 [37].

Bubble column reactors were used for seed culture preparation with 10 L h^−1^ CO_2_/Air (5/95 % (v/v) applying the same irradiation and temperature as before using BG11 medium with 35.0 g L^‐1^ NaCl in a modified *Profors* incubator (Infors, Bottmingen, Switzerland) [38, 39]. After two weeks, the cells were centrifuged (3260 *g*, 15 min; Rotixa 50RS, Hettich, Tuttlingen, Germany) at cell densities of 0.7 ‐ 1.0 g_CDW_ L^‐1^, resuspended in fresh medium and used as inoculum for batch processes in flat‐plate gas‐lift photobioreactors.

### 2.2 Batch cultivations of *D. salina* in flat‐plate gas‐lift reactors

Batch processes were performed in flat‐plate gas‐lift photobioreactors (Labfors Lux 5, Infors HT, Bottmingen, Switzerland) with initial cell dry weights of between 0.2 ‐ 0.3 g_CDW_ L^‐1^. The flat‐plate gas‐lift photobioreactors with a light path length of 2 cm and an LED illuminated surface area of 0.09 m^2^ were operated with a working volume of 1.8 L of modified BG11 media with 35.0 g L^‐1^ NaCl. The differences in composition between BG11 medium and the modified BG11 media are listed in Table 1. Both modified media (“BG11+” and “BG11 Biomass”) contain higher nitrate and phosphate concentrations in order to achieve higher biomass concentrations. In addition, the “BG11 Biomass” medium has increased sulfate and iron concentrations which were added to fulfil elemental balances based on the composition of marine microalgae [40, 41].

**TABLE 1:** Differences in composition of the media “BG11+” and “BG11 Biomass” used in the batch processes compared to BG11 medium applied for seed culture preparation

Component	BG11	BG11+	BG11 biomass
NaCl, g L^‐1^	35/50	35	35
NaNO_3_, mg L^‐1^	1500	6000	3750
K_2_HPO_4_, mg L^‐1^	40	80	240
MgSO_4_ · 7 H_2_O, mg L^‐1^	75	75	600
Ammonium ferric citrate, mg L^‐1^	6	6	12

Mixing and gassing were achieved with 2 NL min^−1^ CO_2_‐enriched sterile air, enabling gas‐lift operation. The CO_2_ content of the inlet gas was varied between 0 and 10 % v/v to control the pH in the photobioreactors.

**FIGURE 1:**
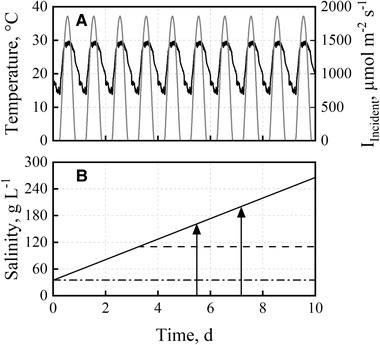
Day‐night cycles of temperature, incident irradiance and salinity applied in batch cultivations of *D. salina* in flat‐plate gas‐lift reactors. (A) Temperature (−) and incident irradiance (−) profile data of June 15, 2012 in Almería, Spain were applied repetitively. (B) The salinity increased from 35 g L^‐1^ NaCl up to 280 g L^‐1^ in 10 days (solid line) and from 35 to 110 g L^‐1^ in 3.3 days and subsequent constant salinity (dashed line) or was maintained at 35 g L^‐1^ NaCl (dashdotted line). The two arrows mark the final salinities of batch cultivations with a shorter process time (160 and 200 g L^‐1^ NaCl)

Temperature and irradiation data from June 15, 2012 in Almería, Spain (Figure 1A) were applied repetitively for all batch cultivation processes with *D. salina*. If not stated otherwise, the temperature ranged between 15 and 30°C, whereas the photosynthetic active incident photon flux density in a wavelength range of 400 to 750 nm was altered from 0 to 1850 μmol m^‐2^ s^‐1^. The light spectrum of the LEDs (Labfors 5 Lux, Infors HT, Bottmingen, Switzerland) was “warm white” and is shown in the supporting information along with the light spectrum ASTM G173‐03 of the sun [42]. Even though spectra of the relative photon flux densities in laboratory and pilot scale differ slightly ([30], compare supporting information), scale‐up was shown to be successful by using the photosynthetic active radiation [29]. Three different salinity profiles were applied, as shown in Figure 1B. The different profiles referred to a reference process at constant seawater salinity (35 g L^‐1^ NaCl), an increase in salinity from 35 to 280 g L^‐1^ NaCl and an increase in salinity up to 110 g L^‐1^ NaCl with subsequently constant salinity. The slope of the salinity increase was 23.1 g L^‐1^ d^−1^ and was estimated based on the compensation of evaporation rates (∼4.2 L m^‐2^) in open thin‐layer cascade photobioreactors (illuminated surface: 8 m^2^, working volume: 50 L) with seawater of 35 g L^‐1^ NaCl [30]. Some batch processes (e.g. pH and temperature variations) were finished earlier, so the salinity at the end of the process resulted in 160 g L^‐1^. As evaporation is highest during the day and relatively low at night, 3.9 g L^‐1^ salt was added six times a day after sampling. Batch processes were generally conducted once. In order to show reproducibility, one batch process (pH 8.5, BG11+‐medium, increased from 35 to 200/280 g L^‐1^) was repeated a second time.

### 2.3 Cell dry weight concentrations and growth rates

The optical density was measured in triplicate six times a day – about every two hours during illuminated conditions – at 750 nm (OD_750_), using a single‐beam spectrometer (Genesys 10S UV‐VIS, Thermo Fisher Scientific Inc., Waltham, USA) with an optical path length of 10 mm. In order to correlate OD_750_ with the cell dry weight concentration, one 5 mL sample of the microalga suspension was taken twice a day. The cells were centrifuged (10 min, 15 000 g), (Rotixa 50RS, Hettich, Tuttlingen, Germany) washed with deionized water, centrifuged again (Mikro 20, Hettich, Tuttlingen, Germany) and dried for 48 h in pre‐dried reaction tubes. OD_750_ and cell dry weight concentrations (c_X_) were linearly correlated for each batch process.

The specific growth rates μ were estimated each day during exponential growth, applying the Matlab R2020a function ‘fit’ of the curve fitting toolbox to identify the parameter μ of Equation 1.

(1) $$c_{x}\left(t\right)={c_{x,}}_{0}\cdot e^{\left(\mu \cdot t\right)}$$

The 95% confidence interval for the growth rate was determined using the function ‘confint’ (Matlab R2017a, Mathworks, Nattick, Massachusetts, USA).

### 2.4 Salinity

The salinity was measured at least twice a day in single determination after salt additions, using a seawater refractometer (HI 96822, Hanna Instruments Deutschland GmbH, Vöhringen, Germany). Samples were diluted to 0–50 g L^‐1^ NaCl.

### 2.5 Extraction and analysis of β‐carotene

The β‐carotene concentration was determined once a day in triplicate while photon flux density was highest. Between 1 and 4 mL of the cell suspension were centrifuged (15 000 g, 10 min; Mikro 20, Hettich, Tuttlingen, Germany) depending on the cell dry weight concentration. The cell pellet was stored at ‐20°C until extraction. The first extraction step was carried out with ∼750 mg glass beads (Ø 0.25‐0.5 mm, Carl Roth GmbH & Co. KG, Karlsruhe, Germany) and 1.0 mL chloroform at 25 Hz for 10 min, using a ball mill (Retsch MM 200, Retsch GmbH, Haan, Germany). After centrifugation (15000 · *g*, 10 min; Mikro 20, Hettich, Tuttlingen, Germany) the supernatant with extracted pigments was collected in reaction tubes and 0.5 mL chloroform added to the remaining cell pellet with the glass beads. The extraction procedure was repeated until the cell pellet and supernatant became colorless. The chloroform with the extracted pigments was allowed to evaporate overnight. After resuspending the extract in 200 μL chloroform, β‐carotene concentration was quantified by HPLC (Dionex Ultimate 3000, Thermo Fischer Scientific, Waltham, Massachussetts, USA) with a Develosil RP‐Aqueous C30 column (particle size: 5 μm, 200 × 4.6 mm, Phenomenex LTD, Aschaffenburg, Germany). The mobile phase consisted of a changing ratio of acetonitrile (A) and chloroform (B): 0–2.5 min: 83% A, 17% B; 2.5‐6 min: 75% A, 25% B; 6–12.5 min: 60% A, 40%B; 12.5‐14 min: 60–83% A, 40‐17% B. Prior to every injection, the system was equilibrated for four minutes. External standards of β‐carotene were used for calibration.

### 2.6 Measurement of incident photon flux density and light transmission

The incident photon flux density and the light transmission were measured with a spectrometer (Flame‐T, Ocean Optics Inc., Florida, USA) in a wavelength range off 400 to 750 nm. To measure the incident photon flux density after passing the glass reactor wall, an identical glass plate was positioned in front of the LEDs at the same distance. The incident photon flux density (μmol m^‐2^ s^‐1^) was measured as a function of the adjustable light intensity of the LEDs (in %) twice a year. The light intensity of the LEDs was adjusted so that the incident photon flux density corresponded to the incident irradiance data from June 15, 2012 in Almería, Spain (Figure 1). The light transmission was measured between one and six times per day at the light‐averted side of the flat‐plate gas‐lift reactor. To minimize scattered light from other light sources in the lab, a black cover – made of silicon with six small openings – was installed, covering the whole surface area of the light‐averted part of the photobioreactor. The light transmission was measured individually at these six defined openings distributed across the black silicon cover, and the mean was used for estimation of light attenuation as a function of biomass concentration.

### 2.7 Light attenuation model

Different light attenuation models like Beer‐Lambert or Reynolds and Pacala [43] were compared (data shown in Supporting Information). A modified Lambert‐Beer model gave the best results for estimating light attenuation caused by *D. salina* suspended in the flat‐plate gas‐lift photobioreactors (see Table 2).

(2) $$I\left(t\right)=I_{0}\;\cdot e^{-\varepsilon \cdot \sqrt{c_{x}}\cdot l}$$

*I(t)* is the measured light transmission at the light‐averted side of the photobioreactor, *I_0_* the incident photon flux density, *ε* the specific extinction coefficient, *c_X_* the time dependent cell dry weight concentration and *l* the length of the light path in suspension (20 mm).

**TABLE 2:** Absorption parameter *ε* of the modified Lambert‐Beer light attenuation model estimated for *D. salina* CCAP 19/18 based on the data of batch processes with “BG11+” and “BG11 Biomass” medium (standard deviation SD, sum of squared errors SSE and coefficient of determination R^2^)

			95% confidence interval			
Medium	ε	SD ε	Lower ε	Upper ε	SSE	R^2^
BG11+	1.40	0.02	1.36	1.45	0.0086	0.9542
BG11 biomass	1.35	0.03	1.30	1.40	0.0174	0.9508

As the incident photon flux density is not constant, a relative photon flux density *I_relative_* was introduced as quotient of transmission and incident photon flux density.

(3) $$I_{\mathrm{rela}\mathrm{tive}}=\frac{I\left(t\right)}{I_{0}}\;=e^{-\varepsilon \cdot \sqrt{c_{x}}\cdot l}\;$$

The specific extinction coefficient *ε* was estimated applying the Matlab R2020a function ‘fit’ of the curve fitting toolbox. The 95 % confidence interval of the specific extinction coefficient was determined using the function ‘confint’ (Matlab R2017a, Mathworks, Nattick, Massachusetts, USA).

## 3 RESULTS AND DISCUSSION

### 3.1 Batch processes with *D. salina* CCAP 19/18 at varying pH

**FIGURE 2:**
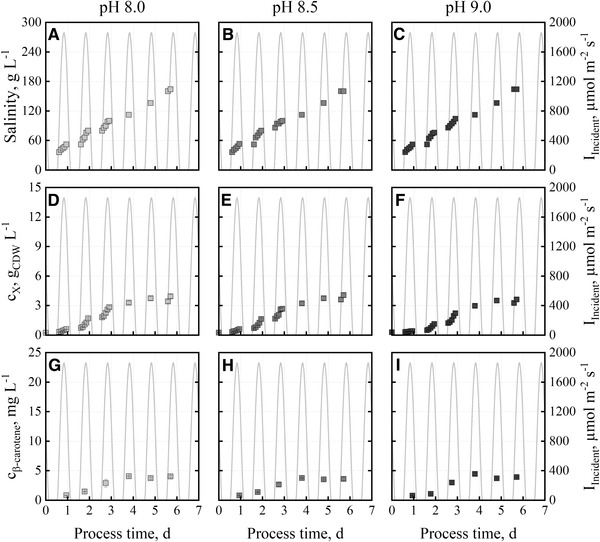
Salinities (A, B, C), cell dry weight concentrations (D, E, F) and β‐carotene concentrations (G, H, I) during batch cultivations of *D. salina* CCAP19/18 with “BG11+” medium in flat‐panel gas‐lift photobioreactors operated at pH 8.0 (light grey), pH 8.5 (grey) and pH 9.0 (dark grey). Temperature (15–30°C) and incident irradiance profile data (0‐1850 μmol m^−2^ s^−1^) of June 15, 2012 in Almería, Spain were applied repetitively

Three batch processes were performed with Mediterranean day‐night cycles of temperature and incident irradiance at varying pH‐setpoints of pH 8.0, pH 8.5, and pH 9.0, respectively (Figure 2) with “BG11+” medium. The salinity was increased by NaCl additions up to 160 g L^‐1^ NaCl after six days to simulate the salinity increase caused by the compensation of evaporation of open thin‐layer cascade photobioreactors with seawater (see section 2.2, evaporation rates from [30]). Final biomass concentrations of 3.6 – 4.1 g_CDW_ L^‐1^ were achieved within 6 days. At pH 9.0, a slightly longer adaptation phase was observed, as the growth rate on day 1 was 0.76±0.11 d^‐1^ compared to 1.98±0.29 d^‐1^ at pH 8.0 and 1.86±0.36 d^‐1^ at pH 8.5. Final β‐carotene concentrations of 3.8 ‐ 4.4 mg L^‐1^ were measured after just 4 days. No significant differences were observed as a function of pH.

Growth of *D. salina* CCAP19/18 was reported at pH 7.5 – pH 8.0 [25, 44]. Other *D. salina* strains showed growth between pH 7.0 – pH 9.18 [34, 45, 46]. We were able to show that *D. salina* CCAP19/18 is able to grow at pH 9.0 as well. As pH should be as high as possible in outdoor cultivations to reduce contamination risks and CO_2_‐loss to the atmosphere – although *D. salina* CCAP19/18 showed an increased lag phase at pH 9.0 – further batch processes were performed at pH 8.5.

### 3.2 Batch processes with *D. salina* CCAP 19/18 at varying temperature profiles

**FIGURE 3:**
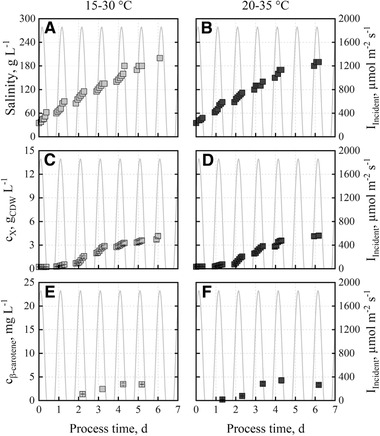
Salinities (A, B), cell dry weight concentrations (C, D) and β‐carotene concentrations (E, F) during batch cultivations of *D. salina* CCAP19/18 with “BG11+” medium in flat‐panel gas‐lift photobioreactors operated at pH 8.5. The repetitively applied temperature profile of June 15, 2012 in Almería, Spain (15–30°C, light grey) was increased by 5°C to 20–35°C (dark grey) without changes in the day‐night dynamics and the incident irradiance profile (0‐1850 μmol m^−2^ s^−1^) of June 15, 2012 (Almería, Spain)

A comparison of growth and β‐carotene production with *D. salina* applying two different temperature profiles was carried out, because outdoor cultivations may be affected by changes in temperature. The repetitively applied temperature profile from June 15, 2012 in Almería, Spain (15‐30°C) was increased by 5°C to 20–35°C without changes in the day‐night dynamics or the incident irradiance profile (0‐1850 μmol m^−2^ s^−1^). Salinity in the “BG11+” medium was increased to 200 g L^‐1^ NaCl. Growth of *D. salina* measured at both temperature levels showed nearly identical trends with similar maximal cell dry weight concentrations (Figure 3). Cell dry weight concentrations of 4.2 g_CDW_ L^‐1^ were measured after 6 days. Final β‐carotene concentrations were measured between 3.5±0.2 mg L^‐1^ at 15–30°C and 4.3±0.6 mg L^‐1^ at 20–35°C. These results indicate that *D. salina* CCAP 19/18 is a robust microalga tolerating temperatures between 15–35°C during day‐night cycles with increasing salinities of up to 110 g L^−1^ NaCl. After reaching this salinity, the cell growth slows down and there was no longer any increase in β‐carotene concentration (Figure 3).

### 3.3 Batch processes with *D. salina* CCAP 19/18 at varying salinity profiles

**FIGURE 4:**
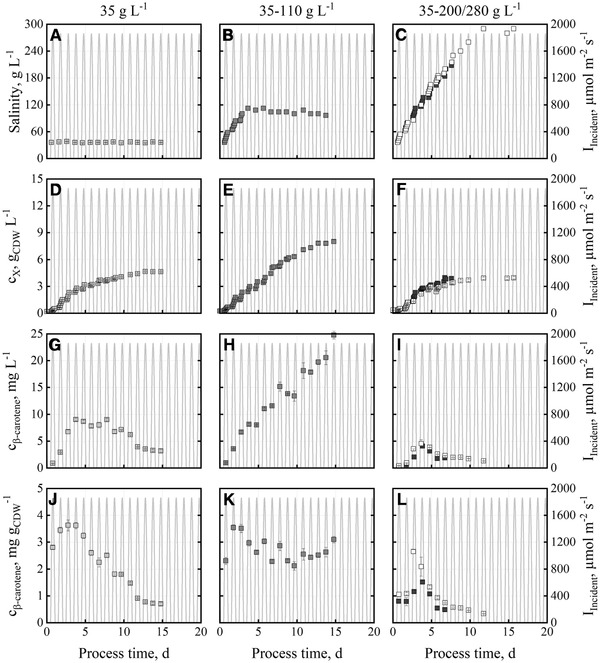
Salinities (A, B, C), cell dry weight concentrations (D, E, F), β‐carotene concentrations (G, H, I) and β‐carotene contents (J, K, L) during batch cultivations with *D. salina* CCAP19/18 in flat‐panel gas‐lift photobioreactors operated at pH 8.5 with “BG11+” medium. The salinity was constant at 35 g L^−1^ (light grey), increased from 35 to 110 g L^−1^ (grey) and increased from 35 to 200/280 g L^−1^ (white, dark grey). Batch processes were carried out applying temperature (15‐30°C) and incident irradiance profile data (0‐1850 μmol m^−2^ s^−1^) of June 15, 2012 in Almería, Spain

Salinity was varied in three different ways (see section 2.2) at pH 8.5 with “BG11+” medium. A maximum cell dry weight concentration of 4.6±0.1 g_CDW_ L^‐1^ was measured at a constant salinity of 35 g L^‐1^ NaCl (Figure 4, left). The β‐carotene concentration reached a maximum of 9.0 mg L^‐1^ (3.6 mg g_CDW_^‐1^) after a process time of 3.8 d. Afterwards the β‐carotene content in dry cell mass decreased until the end of the batch process (< 1 mg g_CDW_^‐1^).

Final algal biomass concentrations of 4.0±0.1 g_CDW_ L^‐1^ were observed in two batch processes with constantly increasing salinity of up to 280 g L^‐1^, and 200 g L^‐1^ (lower salinity due to shorter process time), respectively (Figure 4, right). The β‐carotene concentrations were at a maximum after a process time of 3.7 ‐ 3.8 d as well, although concentrations (4.1±0.1 g L^‐1^ and 4.5±0.7 mg L^‐1^) and β‐carotene content in dry cell mass were reduced (1.5 mg g_CDW_^‐1^ and 2.7 mg g_CDW_^‐1^). Afterwards, the β‐carotene content in algal biomass decreased until the end of the batch process (< 0.5 mg g_CDW_^‐1^). Biomass and β‐carotene concentration show high reproducibility as function of process time (Figure 4, right).

Based on the decreasing β‐carotene content, the constant increase in salinity was stopped at a process time of 3.8 d (Figure 4, middle). Afterwards, the salinity was kept constant at 110 g L^‐1^ NaCl. This resulted in a steadily increasing algal biomass concentration of up to 8.0±0.2 g_CDW_ L^‐1^ after a process time of 14.8 d. The β‐carotene concentration increased as well to final 25 mg L^‐1^ and the β‐carotene content of the algae fluctuated slightly between 2.1 and 3.1 mg g_CDW_^‐1^ until harvest.

Most *D. salina* strains are known to produce β‐carotene at salinities of up to 270 g L^‐1^ NaCl, but growth was reported to stop at salinities between 180 and 210 g L^‐1^ NaCl [19, 47]. Best growth and pigment production of an isolated *D. salina* strain was reported between 88 and 175 g L^‐1^ NaCl [48]. *D. salina* CCAP19/18 was reported to produce the highest amount of carotenoids at the highest salinity tested (116.9 g L^‐1^ NaCl) at a constant temperature of 34°C and incident irradiation of 150 μmol m^−2^ s^−1^ in shaking flasks [24]. The β‐carotene concentration achieved in this study at a maximum salinity of 110 g L^−1^ in flat‐panel gas‐lift bioreactors (25 mg L^‐1^), applying day‐night cycles and temperature profiles of a Mediterranean summer day, was three times higher than the data reported (8.6 mg L^−1^ total carotenoids with no information on β‐carotene concentration or content) [24].

### 3.4 Batch processes with *D. salina* CCAP 19/18 with varying medium compositions

**FIGURE 5:**
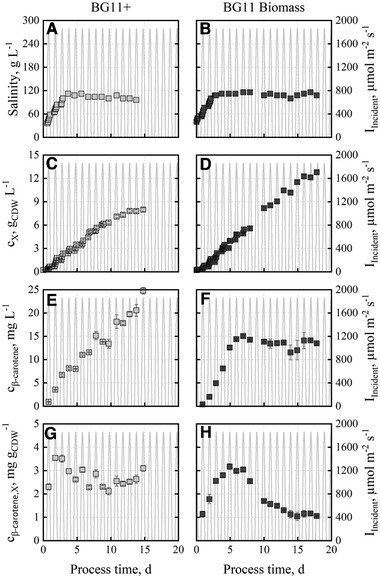
Salinities (A, B), cell dry weight concentrations (C, D), β‐carotene concentrations (E, F) and β‐carotene contents (G, H) during batch cultivations with *D. salina* CCAP19/18 at pH 8.5. The used media were “BG11+” (light grey) and “BG11 Biomass” (dark grey). The salinity was increased from 35 to 110 g L^−1^. Batch processes were carried out at temperature (15‐30°C) and incident irradiance profile data (0‐1850 μmol m^−2^ s^−1^) of June 15, 2012 in Almería, Spain

So far, the highest concentration of up to 8.0±0.2 g_CDW_ L^‐1^ *D. salina* CCAP19/18 was observed in the batch processes with increasing salinity from 35 to 110 g L^−1^ (Figure 4, middle). To avoid any limitation, the medium composition was adapted by increasing the K_2_HPO_4_ by a factor of 3, MgSO_4_ by a factor of 8, and ammonium ferric citrate by a factor of 2 (Table 1). In a batch process, applying the new “BG11 Biomass” medium at pH 8.5 with a constantly increasing salinity up to 110 g L^‐1^ (Figure 5B), a final algal biomass concentration of 12.8±0.2 g_CDW_ L^‐1^ was achieved after a process time of 17.8 d, which corresponds to an increase of 60 % compared to a cultivation with the “BG11+” medium. Compared with data from the relevant literature of laboratory scale cultivations, the final *D. salina* dry weight concentrations were thus increased by a factor of 4.7 to 12.6 [33, 34].

The volumetric biomass productivities of both batch processes remain unchanged within the estimation error (0.77±0.02 g_CDW_ L^‐1^ d^−1^ with “BG11+” and 0.79±0.02 g_CDW_ L^‐1^ d^−1^ with “BG11 Biomass” medium) indicating light limitation. It must be pointed out that these biomass space‐time yields represent a more than 50‐fold increase compared to 15 mg_CDW_ L^‐1^ d^−1^ so far reported with *D. salina* produced in a flat‐plate photobioreactor at photoautotrophic conditions [32]. Applying *D. salina* in a tubular photobioreactor with a CO_2_ supply and 12 h cycles of light and dark [34] resulted in the highest biomass space‐time yield so far reported (0.54 g_CDW_ L^‐1^ d^‐1^), which is 31 % lower than our results.

In contrast to roughly stable β‐carotene contents of 2 to 3 mg g_CDW_^‐1^ in the batch process with the “BG11+” medium (Figure 5A), the β‐carotene contents of the microalgae produced with the new “BG11 Biomass” medium decreased continuously after reaching a maximum of 3 mg g_CDW_^‐1^ at a process time of 5 to 7 d, resulting in a relatively constant β‐carotene concentration of 13.6±0.9 mg L^‐1^ until the end of the batch process. A maximum volumetric β‐carotene productivity of 2.9 mg L^‐1^ d^‐1^ was reached after 2.8 d. The overall volumetric productivity was estimated to be 1.7 mg L^‐1^ d^‐1^. In literature, after applying stress phase of 10 days a comparable overall productivity of 1.7 mg L^‐1^ d^‐1^ was published with a maximum of 1.9 mg L^‐1^ d^‐1^ in shaked Erlenmeyer flasks [49]. Applying a column reactor, an overall volumetric β‐carotene productivity of 5 mg L^‐1^ d^‐1^ was shown [50].

**FIGURE 6:**
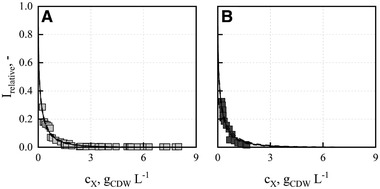
Relative photon flux densities (*I_relative_*) in flat‐panel gas‐lift reactors as function of cell dry weight concentration *c_X_* of *D. salina* CCAP 19/18. The parameter ε (specific extinction coefficient) of the modified Lambert‐Beer light attenuation model (−) was estimated both with the data of the batch processes with the “BG11+” (A, light grey) and “BG11 Biomass” medium (B, dark grey)

One possible reason for the decreasing β‐carotene content with the “BG11 Biomass” medium might be a reduced light availability at higher cell densities, which was previously reported to suppress β‐carotene production [51]. To prove this hypothesis, the relative photon flux densities in the flat‐panel gas‐lift reactors were determined as a function of the cell dry weight concentration of both batch processes with the “BG11+” and “BG11 Biomass” media (Figure 6). The estimated specific absorption coefficients ε of the microalgae in both batch processes are summarized in Table 2.

The relative photon flux density in the suspension approaches zero in both batch processes at cell dry weight concentrations exceeding 3 g_CDW_ L^‐1^, indicating light limitation. 3 g_CDW_ L^‐1^ was measured at a process time of 5 d in both batch processes, and corresponds to the process time at which the maximum β‐carotene content of *D. salina* CCAP 19/18 was observed for the “BG11 Biomass” medium. As light limitation (relative photon flux density approaches zero) was observed at the same process time independently of the medium applied, it can be concluded that (i) a reduced light availability does not suppress β‐carotene production and (ii) nutrient limitation (K_2_HPO_4_, MgSO_4_, and/or ammonium ferric citrate) may have caused stable β‐carotene content in the cells produced with the “BG11+” medium (Figure 5A).

Light limitations in algal cultures can result from high culture depths like in open ponds and high cell densities, as mutual shading between the cells takes place [8]. Due to these limitations, the biomass concentrations observed are not scalable to open pond systems. Other open reactor systems like thin‐layer cascade reactors were already shown to produce high biomass concentrations up to 50 g L^−1^ [30]. Due to their small culture depth (∼ 6 mm) and the resulting higher light availability, light limitations in this reactor system would only occur at higher biomass concentrations compared to our laboratory scale batch processes with a culture depth of 20 mm. Theoretically, even higher biomass concentrations would be possible in thin‐layer cascade photobioreactors.

## 4 CONCLUDING REMARKS

It has been reported that β‐carotene accumulation in *D. salina* can be induced by increasing salinity, increasing photon flux density or limiting nutrients [21, 22, 23]. Increasing the salinity occurs naturally by evaporation in open photobioreactor cultivations of *D. salina* with sea‐water. Increasing the photon flux density in typical day‐night climate conditions can be achieved either by operating photobioreactors at low cell densities, or by reducing the light path in the cell suspension. The operation of a bioreactor at low cell densities may not result in an efficient bioprocess with products accumulating in an intracellular manner (like β‐carotene), due to the resulting low product concentrations. Consequently, day‐night cycles imitating typical Mediterranean climate conditions regarding light and temperature have been applied in this study to investigate growth and β‐carotene production of the strain *D. salina* CCAP19/18 in flat‐plate gas‐lift photobioreactors with a small light path of 2 cm at increasing salinity, simulating the evaporation of open thin‐layer photobioreactors. As a consequence of the reduced light path, high concentrations of *D. salina* CCAP19/18 of up to 8.0 g_CDW_ L^‐1^ were made possible in batch processes within 2 weeks, resulting in a final β‐carotene concentration of 25 mg L^‐1^ (3 mg g^−1^ dry cell weight). The overall volumetric productivity was estimated to be 1.7 mg L^‐1^ d^‐1^ with a maximum volumetric ß ‐carotene productivity (2.9 mg L^‐1^ d^‐1^) after 2.8 d.

Total carotenoid concentrations (including ß‐carotene, lutein, astaxanthin, lycopene) of 19–20 mg L^‐1^ measured spectrometrically have already been reported applying open raceway pond cultivations at typical cell concentrations of 0.33‐1.2 g_CDW_ L^‐1^ *D. salina* (estimated content of 20 to 57 mg carotenoids g^−1^ dry cell weight) [26, 27]. An overall β‐carotene productivity of 1.7 mg L^‐1^ d^‐1^ with a maximum of 1.9 mg L^‐1^ d^‐1^ was published in shaked Erlenmeyer flasks [49]. Applying a column reactor an overall volumetric β‐carotene productivity of 5 mg L^‐1^ d^‐1^ was shown [50].

It seems that the strain *D. salina* CCAP19/18 may not be the best β‐carotene producer, which in addition shows a relatively low tolerance to increasing salinity, as best results were achieved at final salinities of up to 110 g L^−1^ NaCl only. The robustness of this strain shown here with respect to temperature (15‐35°C) and pH (pH 8‐9) is of no advantage if intracellular β‐carotene accumulation capacity and salinity tolerance are limited. The intracellular β‐carotene accumulation capacity of *D. salina* CCAP19/18 was shown to be independent of light availability, but nutrient limitation (K_2_HPO_4_, MgSO_4_, and/or ammonium ferric citrate) seems to enable stable β‐carotene content in the algal cells, despite increasing cell densities in the photobioreactor.

Fully‐controlled parallel lab‐scale photobioreactors like the flat‐panel gas‐lift reactor systems used in this study and the simulation of typical climate conditions of any region of interest (e.g. incident photon flux density profiles, temperature profiles and increasing salinities due to evaporation) are valuable tools to enable a more realistic lab‐scale characterization of microalgae for production processes, projected in open photobioreactor systems.

## CONFLICT OF INTEREST

The authors have declared no conflict of interest.

## Supporting information

Supporting Information

## References

[1] Schenk, P. M. , Thomas‐Hall, S. R. , Stephens, E. , Marx, U. C. et al., Second generation biofuels: high‐efficiency microalgae for biodiesel production. Bioenerg. Res. 2008, 1, 20–43.

[2] Wang, B. , Li, Y. , Wu, N. , Lan, C. Q. , CO bio‐mitigation using microalgae. Appl. Microbiol. Biotechnol. 2008, 79, 707–718. 18483734 10.1007/s00253-008-1518-y

[3] Chiu, S.‐Y. , Kao, C.‐Y. , Chen, C.‐H. , Kuan, T.‐C. et al., Reduction of CO_2_ by a high‐density culture of *Chlorella* sp. in a semicontinuous photobioreactor. Bioresource technol. 2008, 99, 3389–3396. 10.1016/j.biortech.2007.08.013 17904359

[4] Karube, I. , Takeuchi, T. , Barnes, D. J. , Biotechnological reduction of CO_2_ emissions, in:, Advances in biochemical engineering/biotechnology, Vol. 46, Springer‐Verlag, Berlin/Heidelberg 1992, pp. 63–79.

[5] Quinn, J. , de Winter, L. , Bradley, T. , Microalgae bulk growth model with application to industrial scale systems. Bioresource Technol. 2011, 102, 5083–5092. 10.1016/j.biortech.2011.01.019 21324679

[6] Chisti, Y. , Biodiesel from microalgae. Biodiesel from microalgae Biotechnol. Adv. 2007, 25, 294–306. 17350212 10.1016/j.biotechadv.2007.02.001

[7] Mata, T. M. , Martins, A. A. , Caetano, N. S. , Microalgae for biodiesel production and other applications: a review. Renew. Sust. Energ. Rev. 2010, 14, 217–232.

[8] Tredici, M. R. , Photobiology of microalgae mass cultures: Understanding the tools for the next green revolution. Biofuels 2010, 1, 143–162.

[9] Wijffels, R. H. , Barbosa, M. J. , An outlook on microalgal biofuels. Science (New York, N.Y.) 2010, 329, 796–799. 10.1126/science.1189003 20705853

[10] Cantrell, K. B. , Ducey, T. , Ro, K. S. , Hunt, P. G. , Livestock waste‐to‐bioenergy generation opportunities. Bioresource technol. 2008, 99, 7941–7953. 10.1016/j.biortech.2008.02.061 18485701

[11] Hannon, M. , Gimpel, J. , Tran, M. , Rasala, B. et al., Biofuels from algae: challenges and potential. Biofuels 2010, 1, 763–784. 21833344 10.4155/bfs.10.44 PMC3152439

[12] Cohen, G. N. , Biosynthesis of Carotene, Vitamin A, Sterols, Ubiquinones and Menaquinones, in:, Microbial Biochemistry, Springer Netherlands, Dordrecht 2014, pp. 523–538.

[13] Frank, H. A. , Cogdell, R. J. , Carotenoids in photosynthesis. Photochem. and photobiol. 1996, 63, 257–264. 10.1111/j.1751-1097.1996.tb03022.x 8881328

[14] Dufossé, L. , Galaup, P. , Yaron, A. , Arad, S. M. et al., *Microorganisms and microalgae as sources of pigments for food use: a scientific oddity or an industrial reality*?, 2005, 16.

[15] Chidambara Murthy, K. N. , Vanitha, A. , Rajesha, J. , Mahadeva Swamy, M. et al., In vivo antioxidant activity of carotenoids from *Dunaliella salina*‐a green microalga. Life sci. 2005, 76, 1381–1390. 15670617 10.1016/j.lfs.2004.10.015

[16] Woortman, D. V. , Jürgens, S. , Untergehrer, M. , Rechenberger, J. et al., Greener aromatic antioxidants for aviation and beyond. Sustainable Energy Fuels 2020, 4, 2153–2163.

[17] Bogacz‐Radomska, L. , Harasym, J. , β‐Carotene — properties and production methods. Food Quality and Safety 2018, 2, 69–74.

[18] Grand View Research , Beta‐Carotene Market Analysis By Source (Algae, Fruits & Vegetables, & Synthetic), By Application (Food & Beverages, Dietary Supplements, Cosmetics, & Animal Feed) And Segment Forecasts To 2024 2016.

[19] Borowitzka, L. J. , Borowitzka, M. A. , Commercial production of ß‐carotene by *Dunaliella Salina* in open ponds. Bullet of marine sci. 1990, 47, 244–252.

[20] Ben‐Amotz, A. , *Dunaliella* β‐Carotene, in:, Enigmatic Microorganisms and Life in Extreme Environments, Springer Netherlands, Dordrecht 1999, pp. 399–410.

[21] Borowitzka, M. A. , Borowitzka, L. J. , Kessly, D. , Effects of salinity increase on carotenoid accumulation in the green alga *Dunaliella salina* . J Appl Phycol 1990, 2, 111–119.

[22] Loeblich, L. A. , Photosynthesis and pigments influenced by light intensity and salinity in the halophile *Dunaliella salina* (Chlorophyta). J. Mar. Biol. Ass. 1982, 62, 493–508.

[23] Ben‐Amotz, A. , Avron, M. , On the factors which determine massive ß‐carotene accumulation in the halotolerant alga *Dunaliella bardawil* . Plant physiol. 1983, 72, 593–597. 16663050 10.1104/pp.72.3.593 PMC1066285

[24] Fazeli, M. R. , Tofighi, H. , Samadi, N. , Jamalifar, H. et al., Carotenoids accumulation by *Dunaliella tertiolecta* (Lake Urmia isolate) and *Dunaliella salina* (CCAP 19/18 & WT) under stress conditions. DARU Journal of Pharmaceutical Sciences 2006, 14, 146–150.

[25] Mojaat, M. , Pruvost, J. , Foucault, A. , Legrand, J. , Effect of organic carbon sources and Fe^2+^ ions on growth and β‐carotene accumulation by *Dunaliella salina* . Biochemical Engineering Journal 2008, 39, 177–184.

[26] Borovkov, A. B. , Gudvilovich, I. N. , Avsiyan, A. L. , Memetshaeva, N. O. A. et al., Production characteristics of Dunaliella salina at two‐phase pilot cultivation (Crimea). Turk. J. Fish. Aquat. Sci. 2020, 20.

[27] Wu, Z. , Dejtisakdi, W. , Kermanee, P. , Ma, C. et al., Outdoor cultivation of *Dunaliella salina* KU 11 using brine and saline lake water with raceway ponds in northeastern Thailand. Biotechnol. and appl. Biochem. 2017, 64, 938–943. 27696529 10.1002/bab.1537

[28] Soratana, K. , Harper Jr., W. F. , Landis, A. E. , Microalgal biodiesel and the renewable fuel standard's greenhouse gas requirement. Energy Policy 2012, 46, 498–510.

[29] Pfaffinger, C. E. , Severin, T. S. , Apel, A. C. , Göbel, J. et al., Light‐dependent growth kinetics enable scale‐up of well‐mixed phototrophic bioprocesses in different types of photobioreactors. J. biotechnol. 2019, 297, 41–48. 30898687 10.1016/j.jbiotec.2019.03.003

[30] Apel, A. C. , Pfaffinger, C. E. , Basedahl, N. , Mittwollen, N. et al., Open thin‐layer cascade reactors for saline microalgae production evaluated in a physically simulated Mediterranean summer climate. Algal Res. 2017, 25, 381–390.

[31] Lamers, P. P. , Janssen, M. , de Vos, R. C. H. , Bino, R. J. et al., Carotenoid and fatty acid metabolism in nitrogen‐starved *Dunaliella salina*, a unicellular green microalga. J. biotechnol. 2012, 162, 21–27. 22750089 10.1016/j.jbiotec.2012.04.018

[32] Khadim, S. R. , Singh, P. , Singh, A. K. , Tiwari, A. et al., Mass cultivation of *Dunaliella salina* in a flat plate photobioreactor and its effective harvesting. Biores. Technol. 2018, 270, 20–29. 10.1016/j.biortech.2018.08.071 30208357

[33] Morowvat, M. H. , Ghasemi, Y. , Culture medium optimization for enhanced β‐carotene and biomass production by *Dunaliella salina* in mixotrophic culture. Biocatal. Agric. Biotechnol. 2016, 7, 217–223.

[34] Kim, W. , Park, J. M. , Gim, G. H. , Jeong, S.‐H. et al., Optimization of culture conditions and comparison of biomass productivity of three green algae. Bioprocess and biosys. eng. 2012, 35, 19–27. 10.1007/s00449-011-0612-1 21909669

[35] Roopnarain, A. , Gray, V. M. , Sym, S. D. , Phosphorus limitation and starvation effects on cell growth and lipid accumulation in *Isochrysis galbana* U4 for biodiesel production. Bioresource technol. 2014, 156, 408–411. 10.1016/j.biortech.2014.01.092 24534441

[36] Wongsnansilp, T. , Juntawong, N. , Wu, Z. , Effects of phosphorus on the growth and chlorophyll fluorescence of a *Dunaliella salina* strain isolated from saline soil under nitrate limitation. J. Biol. Res. 2016, 89.

[37] Waterbury, J. B. , Stanier, R. Y. , Isolation and growth of cyanobacteria from marine and hypersaline environments, in:, The Prokaryotes, Springer Berlin Heidelberg, Berlin, Heidelberg 1981, pp. 221–223.

[38] Altenbach‐Rehm, J. , Nell, C. , Arnold, M. , Weuster‐Botz, D. , Parallel bubble columns with fed‐batch technique for microbial process development on a small scale. Chem. Eng. Technol. 1999, 22, 1051–1058.

[39] Havel, J. , Franco‐Lara, E. , Weuster‐Botz, D. , A parallel bubble column system for the cultivation of phototrophic microorganisms. Biotechnol. letters 2008, 30, 1197–1200. 10.1007/s10529-008-9680-y 18488151

[40] Grobbelaar, J. U. , Inorganic algal nutrition, in:, Handbook of Microalgal Culture, John Wiley & Sons, Ltd, Oxford, UK 2013, pp. 123–133.

[41] Barsanti, L. , Gualtieri, P. , Algae: Anatomy, Biochemistry, and Biotechnology. A CRC Press book, CRC Press, Boca Raton, Fla 2006.

[42] NREL, Terrestrial Reference Spectra for Photovoltaic Performance Evaluation (ASTM G173–03) [Internet] [cited 2020 Aug 4]. Available from: https://www.nrel.gov/grid/solar-resource/spectra-am1.5.html.

[43] Reynolds, H. L. , Pacala, S. W. , An analytical treatment of root‐to‐shoot ratio and plant competition for soil nutrient and light. The American naturalist 1993, 141, 51–70. 10.1086/285460 19426022

[44] Nguyen, S. , Tran, D. , Portilla, S. , Vo, T. , Medium improvement for higher growth and longer stationary phase of *Dunaliella* . J. Plant Sci. 2014, 2, 9–13.

[45] Abu‐Rezq, T. S. , Al‐Hooti, S. , Jacob, D. A. , Optimum culture conditions required for the locally isolated *Dunaliella salina* . J. algal biomass utilization 2010, 1, 12–19.

[46] Ying, K. , Effects of CO_2_ and pH on Growth of the Microalga *Dunaliella salina* . J. Microb. Biochem. Technol. 2014, 06.

[47] Borowitzka, L. J. , Borowitzka, M. A. , Moulton, T. P. , The mass culture of *Dunaliella salina* for fine chemicals: From laboratory to pilot plant. Hydrobiologia 1984, 116–117, 115–121.

[48] Farhat, N. , Rabhi, M. , Falleh, H. , Jouini, J. et al., Optimization of salt concentrations for a higher carotenoid production in *Dunaliella salina* (Chlorophyeae). J. phycol. 2011, 47, 1072–1077. 27020189 10.1111/j.1529-8817.2011.01036.x

[49] Shaker, S. , Morowvat, M. H. , Ghasemi, Y. , Effects of sulfur, iron and manganese starvation on growth, β‐carotene production and lipid profile of *Dunaliella salina* . JYP 2017, 9, 43–46.

[50] Wu, Z. , Duangmanee, P. , Zhao, P. , Juntawong, N. et al., The effects of light, temperature, and nutrition on growth and pigment accumulation of three *Dunaliella salina* strains isolated from saline soil. Jundishapur J. Microbiol. 2016, 9, e26732. 27099682 10.5812/jjm.26732 PMC4833956

[51] Lamers, P. P. , van de Laak, C. C. W. , Kaasenbrood, P. S. , Lorier, J. et al., Carotenoid and fatty acid metabolism in light‐stressed *Dunaliella salina* . Biotechnol. Bioeng. 2010, 106, 638–648. 20229508 10.1002/bit.22725

