# Supplementary material for: Production of β‐carotene with Dunaliella salina CCAP19/18 at physically simulated outdoor conditions
Source: Eng Life Sci. 2020 Sep 13;21(3-4):115–25. doi: 10.1002/elsc.202000044 (PMC7923581; doi:10.1002/elsc.202000044)
Supplement: Supplementary file 1 — Supporting Information [file ELSC-21-115-s001.pdf]

Production of β-carotene with *Dunaliella salina* CCAP19/18 at Physically Simulated Outdoor Conditions - Supporting Information:

(Lara Wolf, Thomas Cummings, Katharina Müller, Manfred Reppke, Marianne Volkmar, Dirk Weuster-Botz)

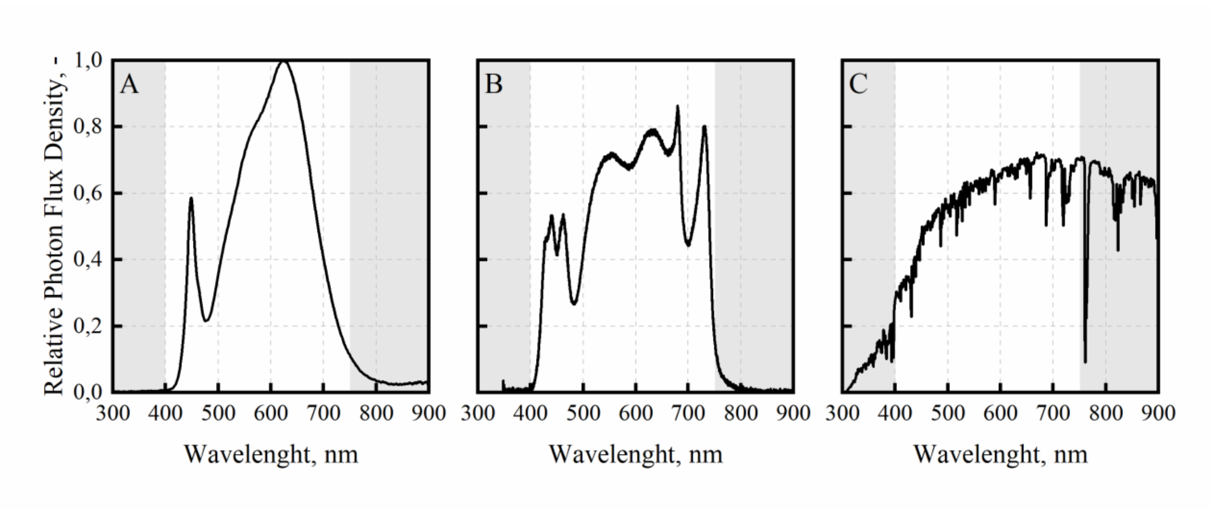

Figure A1: Comparison of different photon flux densities. The normalization of the spectra was conducted with reference to the same photosynthetic active irradiation (wavelengths 400-750 nm, white area). A: spectrum of warm white LEDs (Labfors 5 Lux, Infors HT, Bottmingen, Switzerland) used in laboratory scale; B: spectrum of LEDs in the TUM AlgaeTec Center [30] and C: solar reference spectrum ASTM G173-03 [43]. In the AlgaeTec Center a combination of sunlight and the shown LED system is used to mimic realistic outdoor light conditions [30].

Table A1: Absorption parameters *ε* of different light attenuation models estimated for *D. salina* CCAP19/18 based on the data of batch processes with "BG11+" and "BG11 Biomass" medium (standard deviation SD, sum of squared errors SSE and coefficient of determination R^2). The compared models were Beer-Lambert, a modified Lambert-Beer, and Reynolds and Pacala.

				95% confidence interval			
Conditions	Modell	ε	SD	lower ε	upper ε	SSE	R^2
"BG11+"	Lambert Beer	2,29	0,10	2,10	2,48	0,0385	0,7939
pH 8.5	mod. Lambert-Beer	1,40	0,02	1,36	1,45	0,0086	0,9542
3.5-110 g L^{-1} NaCl	Reynolds-Pacala	6,72	0,36	6,01	7,43	0,0178	0,9049
"BG11 Biomass"	Lambert Beer	2,28	0,05	2,17	2,38	0,0197	0,9443
pH 8.5	mod. Lambert-Beer	1,35	0,03	1,30	1,40	0,0174	0,9508
3.5-110 g L^{-1} NaCl	Reynolds-Pacala	6,01	0,36	5,31	6,70	0,0374	0,8943
